# Operative Treatment of Dislocated Shoulder

**Published:** 1890-02-15

**Authors:** 


### OPERATIVE TREATMENT OF DISLOCATED SHOULDER.

Sir Joseph Lister contributes to a contemporary two cases of long-standing dislocation of both shoulders treated by operation. Sir Joseph remarks that he once had the misfortune to cause the death of a patient from rupture of the axillary artery in an attempt to reduce a dislocated shoulder. But it would seem that the patient was more to blame than the operator, for the dislocation must have been of older date than represented. And of course the success of the ordinary method of reducing a dislocation depends very much on the time the bone has been "out of joint." The first case now reported by Sir Joseph Lister is that of a labourer who, from a fall of forty feet, sustained subcoracoid dislocation of both shoulders. Eight weeks afterwards, instead of pursuing the usual course, Sir Joseph Lister operated as follows: Having made an incision from the coracoid process downwards and somewhat outwards, in the interval between the deltoid and pectoralis major, Sir Joseph divided the tendon of the subscapularis muscle at its insertion, and then with a periosteum detacher separated the soft parts from the head and inner part of the neck of the bone. Pulleys were then applied, and some fibrous bands put on the stretch were divided. But the head of the humerus would not return to the normal position. Therefore the head of the bone was protruded as if for resection, and the external rotators cut through at their insertions. Pulleys were again applied, and after a time the bone slipped into position. All now went on favourably. On the following week the other shoulder was operated on in similar manner, except that guided by experience, the head of the bone was at once protruded and the attachments of the rotators divided. Five months afterwards the patient put on his coat and waistcoat unaided. The patient now states he can do any hard agricultural work as well as ever, but cannot elevate the limbs above the horizontal level. The second case was one of dislocation of both shoulders during an epileptic fit, into the subcoracoid position. He was operated upon in the same manner as the former patient (left shoulder) about seven and a-half months after the dislocation, and the results, although distinctly successful, were not brilliant. After six months, however, the patient besought operation on the other side. Sir Joseph decided that instead of detaching the soft parts from the end of the humerus and attempting reduction, he would cut down on the head of the bone and remove it by hammer and chisel without disturbing the external rotators. "For a study of the skeleton with the humerus in the subcoracoid position had shown me that the removal of the articular portion without interfering with the tuberosities would allow the bone to drop back into relation with the glenoid cavity." This was done, and answered entirely. Ultimately the patient gained nearly full use of his arms and shoulders, but could not raise them into the horizontal position. From the fact that he is now able to gain his living on a farm, it may be inferred that the treatment was a great success, which a recurrence of an epileptic fit does not appear to have jeopardised. Sir Joseph Lister considers that the result of these two cases is encouraging, and that by such means injury to the axillary vessels will be avoided. Something of the success of the treatment may be attributed to the antiseptic dressings, and precautions employed. The operations as above may be regarded as an entirely new feature in surgery, and certainly the success attained warrants recourse to similar measures in old standing cases of dislocation.

